# Interindividual variation of progesterone elevation post LH rise: implications for natural cycle frozen embryo transfers in the individualized medicine era

**DOI:** 10.1186/s12958-023-01096-4

**Published:** 2023-05-18

**Authors:** Carol Coughlan, Baris Ata, Raquel Del Gallego, Barbara Lawrenz, Laura Melado, Suzan Samir, Human Fatemi

**Affiliations:** 1 ART Fertility Clinic, Dubai, UAE; 2 ART Fertility Clinic, Abu Dhabi, UAE; 3 Faculty of Medicine, Department of Obstetrics & Gynecology, Koc University, Istanbul, Turkey; 4 Women’s University Hospital, Tuebingen, Germany

**Keywords:** Progesterone, Luteinizing hormone, Ovulation, Assisted reproduction, Menstrual cycle

## Abstract

**Background:**

The key to optimal timing of frozen embryo transfer (FET ) is to synchronize the embryo with the receptive phase of the endometrium. Secretory transformation of the endometrium is induced by progesterone. In contrast, detection of the luteinizing hormone (LH) surge is the most common surrogate used to determine the start of secretory transformation and to schedule FET in a natural cycle. The accuracy of LH monitoring to schedule FET in a natural cycle relies heavily on the assumption that the period between the LH surge and ovulation is acceptably constant. This study will determine the period between LH rise and progesterone rise in ovulatory natural menstrual cycles.

**Methods:**

Retrospective observational study including 102 women who underwent ultrasound and endocrine monitoring for a frozen embryo transfer in a natural cycle. All women had serum LH, estradiol and progesterone levels measured on three consecutive days until (including) the day of ovulation defined with serum progesterone level exceeding 1ng/ml.

**Results:**

Twenty-one (20.6%) women had the LH rise 2 days prior to progesterone rise, 71 (69.6%) had on the day immediately preceding progesterone rise and 10 (9.8%) on the same day of progesterone rise. Women who had LH rise 2 days prior to progesterone rise had significantly higher body mass index and significantly lower serum AMH levels than women who had LH rise on the same day with progesterone rise.

**Conclusion:**

This study provides an unbiased account of the temporal relationship between LH and progesterone increase in a natural menstrual cycle. Variation in the period between LH rise and progesterone rise in ovulatory cycles likely has implications for the choice of marker for the start of secretory transformation in frozen embryo transfer cycles. The study participants are representative of the relevant population of women undergoing frozen embryo transfer in a natural cycle.

## Introduction

The advent of vitrification and excellent cryo-survival rates, increasing elective single embryo transfers and preimplantation genetic testing cycles contributed to increasing use of frozen embryo transfers (FET), which now comprise the majority of embryo transfers in many countries. While FET can be done in an artificial or natural cycle (NC) the latter is gaining momentum as the preferred option for frozen embryo transfer (FET) driven by studies suggesting that livebirth rates are higher with improved maternal, obstetric and perinatal outcomes as compared to hormonal replacement (HRT) FET cycles [1–7].

The key to optimal timing of FET is to synchronize the embryo with the receptive phase of the endometrium. Secretory transformation of the endometrium is induced by progesterone [8] and in artificial cycles (AC), FET is scheduled according to the start of exogenous progesterone, i.e., a blastocyst is ideally transferred on the 6th day of progesterone exposure [9]. In contrast, detection of the luteinizing hormone (LH) surge is the most common surrogate used to determine the start of secretory transformation and to schedule FET in a natural cycle. Urinary LH kits are often used in a NC FET [10]. The accuracy of LH monitoring to schedule FET in a natural cycle relies heavily on the assumption that the period between the LH surge and ovulation, i.e., progesterone rise and the start of secretory transformation, is acceptably constant.

It is a well-recognized phenomenon that the endocrine profile of menstrual cycles varies not only amongst women but also from cycle to cycle for any given female [11–13]. In research as in clinical practice, when describing the menstrual cycle the average of individual hormonal profiles is taken as normal with an accepted “normal” range without taking into consideration inter-cycle variability [10]. It has been demonstrated that most hormone trajectories in individual women differ considerably from the mean hormonal curves and LH surges culminating in ovulation appear to be highly variable in timing, amplitude and duration [14–17]. Moreover, secretory transformation of the endometrium and preparation for the window of implantation is provided by progesterone exposure and, not LH per se [8]. Hence, the timing of LH is used as an indirect marker of the timing of progesterone rise and the start of the secretory transformation to schedule FETs. Given the variation in the endocrine profile of a NC, LH may not serve as the best benchmark to schedule FET.

The present study investigates whether the time period between the LH rise and progesterone rise varies by employing both ultrasound monitoring of follicular growth and serial hormonal profile assessment in natural ovulatory menstrual cycles.

## Materials and methods

Anonymized electronic medical records of all natural FET cycles conducted at a tertiary referral fertility clinic between 1st January 2017 and 31st August 2021 were screened retrospectively. Institutional local Ethical Committee approved the study protocol (REFA077).

Inclusion criteria were age 18 years to 43 years with regular menses between 26 and 34 days, a body mass index (BMI) between 18 and 35 kg/m^2^, having at least one euploid blastocyst available for transfer, and measurements of serum estradiol, LH and progesterone levels on three consecutive days until (including) the day of presumed ovulation as defined with a serum progesterone level ≥ 1 ng/ml [8]. Each woman was included only with one cycle.

### Monitoring natural cycle

**Fig. 1:**
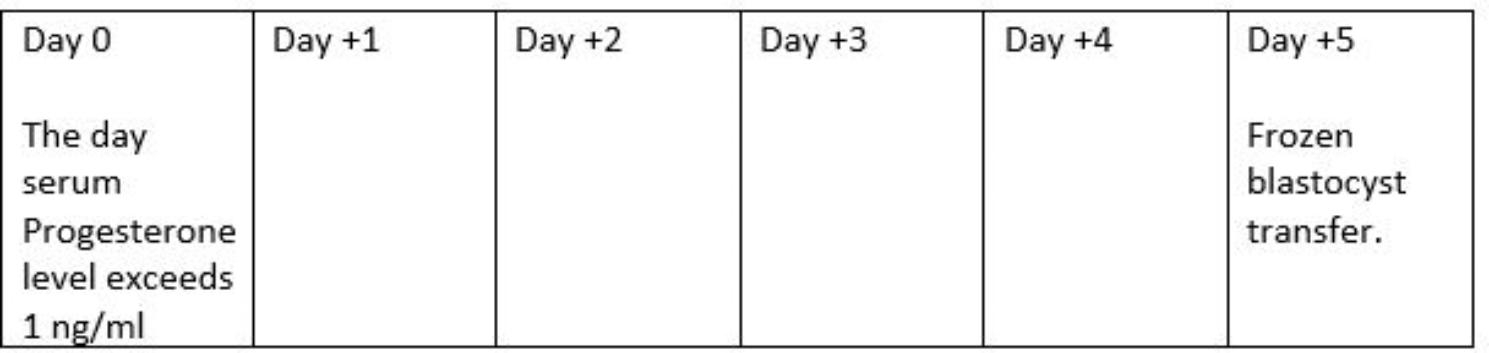
Scheduling frozen blastocyst transfer based on serum progesterone rise

Commencing on the second day of menses and intermittently throughout the patients’ NC ultrasound scans were performed to monitor follicular growth. Serial measurements of serum LH, FSH, estradiol and progesterone levels were commenced once a dominant follicle measuring ≥ 14 mm was identified to accurately determine the time of ovulation. The LH surge was considered to have begun when the concentration rose by 180% above the most recent serum value and continued to rise thereafter [18–20]. Progesterone concentrations of 1.0ng/ml and above were regarded as the confirmation for occurrence of ovulation, and this was designated as day 0, to schedule embryo transfer on the fifth day after progesterone rise, e.g., if progesterone rise was on a Monday, blastocyst transfer would be on Saturday (Fig. 1) [8, 18–20].

All blood samples used for this analysis were collected between 9am and 12am and assayed at ART Clinical Laboratory, Abu Dhabi, with an automated Elecsys® immunoanalyzer (Roche Diagnostics, Mannheim, Germany).

ELECSYS® progesterone generation III assay was used to measure progesterone. The measuring range is 0.47-56.4ng/ml as per in -house validation). The Elecsys Estradiol III assay has a measuring range of 21.2-2593pg/mL (In-house validation). The Elecsys LH assay shows negligible cross reactivity with FSH, TSH, hCG, hGH, and hPL. The measuring range is 0.99-197mIU/mL (ART Clinical Laboratory In-house validation).

### Statistical analysis

Continuous variables were defined with mean and standard deviation or median and quartiles depending on distribution characteristics. Categorical variables were defined with numbers and percentages.

Serum hormone levels were plotted across menstrual cycle days. Graphs were centralized on the designated “day 0”, i.e., the day of progesterone rise over 1 ng/ml.

Comparison of patient characteristics among women with different intervals between the day of the LH surge and day 0 were with t-test, Mann-Whitney U, or derivatives of chi-squared tests determined by variable and distribution characteristics.

## Results

**Fig. 2:**
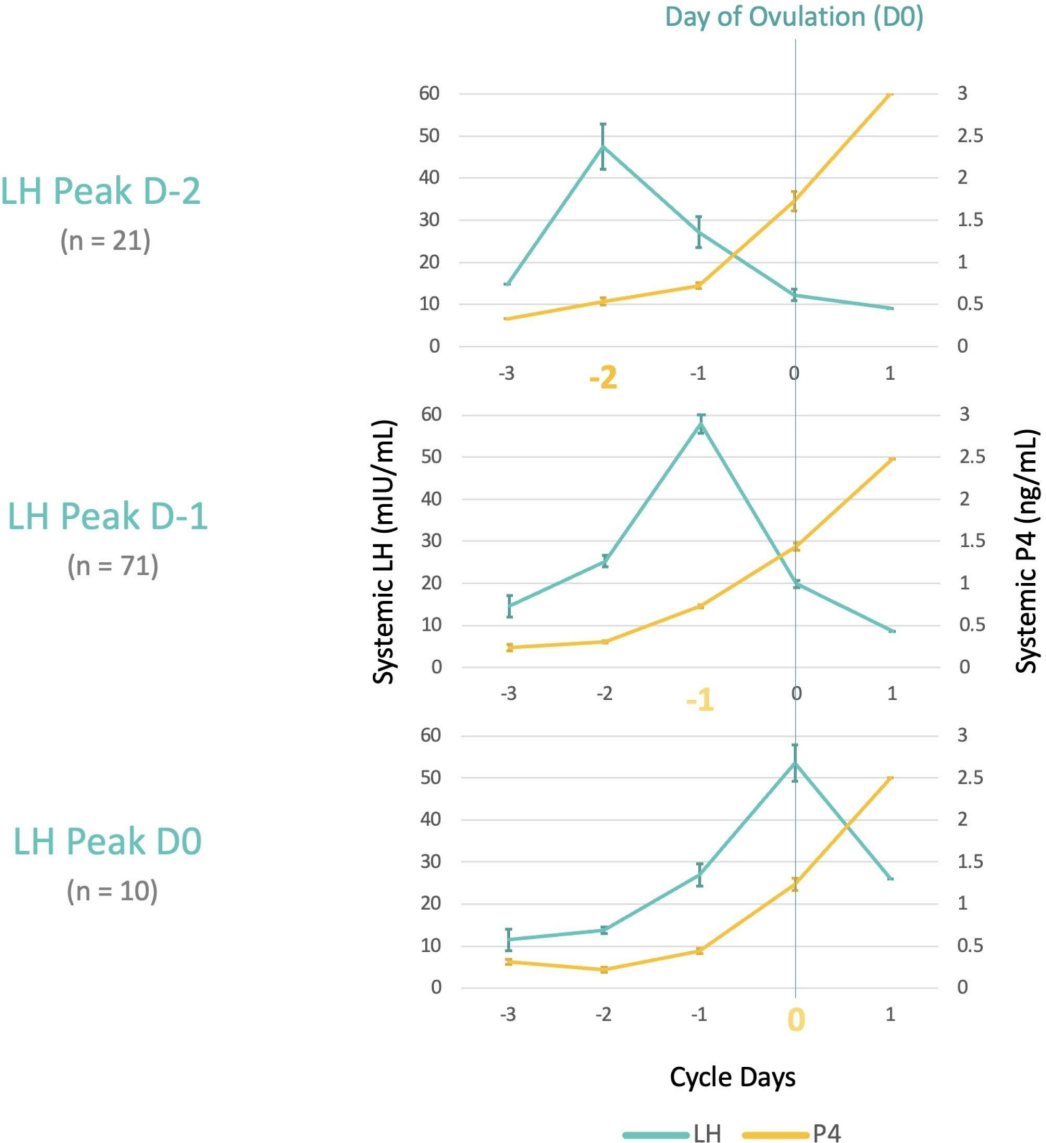
Graphic representation of the observed different periods between LH peak and progesterone rise

102 women had serial hormonal measurements on the three consecutive days ending in ovulation, day − 2, day − 1 and day 0 (the day of confirmed ovulation by serum progesterone levels ≥ 1 ng/ml). Three distinct patterns of ovulation, defined with the period between LH rise and progesterone rise were identified among these 102 women. Twenty-one (20.6%) women had the LH rise 2 days prior to ovulation (day − 2), 71 (69.6%) had on the day immediately preceding ovulation (day − 1) and 10 (9.8%) attained the LH peak on the day of ovulation (Day 0) (Fig. 2).

Based on these observations, if FET was scheduled based on LH rise, 30.4% of the patients would have their FET scheduled on a different day than FET scheduled according to progesterone rise, since the period between LH rise and progesterone rise would be approximately one day longer in 20.6% of the participants and one day shorter in 9.8%, than the anticipated 24 h period.

While women with LH rise on different days in relation to ovulation were similarly aged, women who had LH rise 2 days before ovulation had significantly higher BMI and significantly lower AMH than women who had LH rise on the same day with ovulation (Table 1).

**Table 1:** Characteristics of women who had different periods between LH rise and progesterone rise

	LH peak on day 0	LH peak on day − 1	LH peak on day − 2
**Age in years**	32.80 ± 4.85	35.31 ± 4.92	34.71 ± 4.88
**AMH in ng/ml**	2.67 (1.83–6.63)^a^	2.14 (1.32–3.46)	1.59 (0.82–2.09)^a^
**BMI in kg/m** ^**2**^	22.39 (20.91–24.96)^b^	25.39 (23.11–30.69)	26.91 (23.74–30.75)^b^

LH, luteinizing hormone; AMH, Anti Mullerian Hormone; BMI, Body mass index. Values are mean ± standard deviation or median (25th – 75th percentile). Values with the same superscript are significantly different with p values of 0.04^a^ and 0.03^b^, both with Bonferonni adjustment

## Discussion

Using a combination of ultrasound monitoring and serial hormonal profiling three distinct patterns of LH rise were identified in relation to ovulation defined as increasing serum progesterone levels ≥ 1 ng/ml corroborating the phenomenon of cycle variability.

Alliende prospectively studied 82 cycles of 25 women [14]. Participants were proven fertile, had ovulation documented by the Billing ovulation peak method [21, 22] and biphasic basal body (BBT) temperature pattern in the previous six cycles before recruitment, and all the 82 cycles studied were also ovulatory as documented by the same two methods. The participants collected early morning urine samples starting from the first day of the cycle, for analyses of estrone glucuronide (EG), LH and pregnanediol glucuronide (PG) levels with noncompetitive radioimmunoassay. In 77% of the 82 cycles at least one hormone displayed a different pattern than the mean curves. In particular, LH pattern was different than the mean curve in 56% of the cycles. The LH peak was defined as “the highest LH numerical value, close to the EG peak and the PG increase”. Importantly, small LH peaks, defined as “LH surge smaller than LH peak, before or after the LH peak; also close to the EG peak and the PG increase” were detected in 19% of the cycles. Importantly, clinical periovulatory indicators (ovulation method peak and BBT) were associated with small LH peaks rather than the LH peak proper in 60% of cycles with small LH peaks. Since these small LH peaks were lower than the LH peak proper, by 29 IU/L/24 h on average (range 11–52 IU/L), it is possible that some may go unrecognized by commercially available urinary LH kits, leading to missing the time when progesterone starts to increase, hence the start of secretory transformation. Moreover, pre-peak and post-peak LH surges, defined as “LH surge is not close to the EG peak and the PG increase” were identified in 22% of the cycles. Such pre-peak LH surges can also lead to incorrect estimation of the time of ovulation and progesterone increase and the start of secretory transformation, with implications for timing of FET in a NC. These findings are on par with our present findings and others which also demonstrated variation between individual women’s hormonal curves and mean curves generated based on aggregate data [14–17]. Collectively these observations suggest that LH per se may not be the best indicator of the time of ovulation, as determined by progesterone rise.

In contrast, a 1980 study included 107 women during the periovulatory period, who provided serial blood samples and the mature follicle or the corpus luteum was removed at laparotomy for histologic examination in order to investigate temporal relationships between ovulation and defined changes in the plasma concentrations of estradiol, LH, follicle stimulating hormone, and progesterone [11]. Based on a statistical model, the concentration of circulating LH was reported to be the best indirect parameter of ovulation at a given time in relation to the interval from a defined rise or peak in the concentration of a circulating hormone. The estimated median time interval (95% confidence intervals) from the LH rise was 32.0 (23.6 to 38.2) hours and from the LH peak was 16.5 (9.5 to 23.0) hours by the model. The final model for LH suggested that it was possible to estimate in only 90% of the cases that ovulation had occurred between 16 (± 6) and 48 (± 6) hours after the first significant rise in the LH concentration and between − 3 (± 5) and 36 (± 5) hours after the LH peak. However, an examination of the results of individual women showed corresponding ranges of between 24 and 56 h from the first significant rise in LH and between 8 and 40 h after the peak. While these results, which show variation by more than a day, could have been viewed as the best available estimates 42 years ago, they can be regarded as inaccurate in today’s individualized medicine era. The ranges of the periods between changes in LH concentration, i.e., either the first increase or the peak, and ovulation in individual women’s data extend over 24 h, which suggests that if FET is scheduled solely based on LH concentrations, in some women, a blastocyst could be transferred on a different day than the targeted 5th day after ovulation, i.e., start of secretory transformation, leading to lower implantation or higher miscarriage rates [23]. Indeed, our observation of varying periods between LH rise and progesterone rise confirm that such an error is possible in daily practice if FET is scheduled based on change in LH concentrations.

Other limitations of currently used LH tests, in particular urinary LH kits, which are commonly used in daily practice for scheduling FET include false negativity, delay in urinary LH rise by nearly 24 h after serum LH rise due to urinary clearance of LH, varying period between detection of urinary LH and observation of follicular collapse by ultrasound, and even seasonal variation in timing of LH detection in urine samples [1, 20, 24–26]. In addition urinary LH kits may demonstrate LH surges in the absence of ovulation [27, 28]. Notably a high percentage of normally cycling women may demonstrate premature LH surges which do not trigger ovulation [28]. Moreover, a marked inter-personal variation in the time interval between the onset of the detected urine LH surge and ovulation has been seen, ranging from 22 to 56 h [10].

Ambiguity surrounds the definition of an LH surge, even if serum LH levels are monitored rather than urinary levels. Various values from 1.8 to 6 fold increase in LH levels from the baseline LH measurements have been used to define the onset of the LH surge, and even the definition of baseline LH level used as the benchmark for the fold increase varies in the literature [10, 15, 17, 19, 29–34]. Absolute value based definitions of the LH surge also vary, e.g., the attainment of LH ≥ 17 mIU/ml during the follicular phase with a ≥ 30% drop in E2 levels the following day [18]; LH > 10 mIU/ml [35]; LH > 15 mIU/ml [36] and LH > 20mIU/ml [37, 38].

While ultrasound monitoring of follicle growth and collapse can be considered as another method of determining the time of ovulation, the issue with using ultrasound confirmation of dominant follicle disappearance as the sole criterion for defining “ovulation” lies in the fact that different patterns of follicular fluid evacuation during ovulation have been described. It is important to note, that in some cases fluid remains in the former follicle even into the luteal phase [39]. As such the detection of dominant follicle disappearance in isolation may not be the most reliable sign of ovulation. The key to optimal timing of frozen embryo transfer is to synchronise the embryo with the receptive phase of the endometrium. We prefer to use the rise in progesterone, which initiates secretory transformation, as the benchmark for timing of embryo transfer in a natural cycle.

Data on the efficacy and timing of embryo transfer in “true“ NC-FET are limited. Previous studies have focused for the most part on modified natural cycles using human chorionic gonadotrophin to induce ovulation of the dominant follicle which has been shown to be inferior to a spontaneous natural cycle for planning of frozen- thawed embryo transfer [19, 40]. Administration of hCG in the late follicular phase induces changes in the endometrium, which would have occurred several days later in a natural cycle [19]. In addition, human chorionic gonadotrophin (hCG) and LH act on the endometrium through the same receptor and it has been suggested that their simultaneous presence may have an adverse effect on pregnancy rates [19, 41].

The observational design of the current study can be considered a limitation of the present work, yet this is a descriptive study and there have been no interventions that can affect serum hormone levels. As such we believe our observations represent an unbiased account of the temporal relationship between LH and progesterone increase in a natural menstrual cycle, in a relevant population of women who were undergoing a NC FET, to whom the results are meant to be extrapolated.

While large fluctuations in serum progesterone levels due to the pulsatile secretion by the corpus luteum can render once daily measurement less reliable in the luteal phase, the major source of progesterone in the follicular phase is from the adrenal gland and the range of fluctuation is very low [42]. Since monitoring ends when progesterone levels exceed 1 ng/ml, we do not think the limited fluctuation in serum progesterone would significantly effect the identification of preovulatory serum progesterone rise.

Since we always scheduled FET according to serum progesterone but not to LH levels, our study does not provide a direct answer to the question whether FET scheduled according to serum progesterone levels provides higher live birth rates than FET scheduled based on urinary or serum LH levels in a natural cycle. Yet, given the above concerns and the three clearly different temporal relationships between LH and progesterone rise observed in our series collectively suggest that scheduling FET according to serum progesterone levels per se rather than its imperfect indirect surrogate LH levels can be a better choice in the current individualized medicine era. Undeniably, the initiation of the secretory transformation of the endometrium and preparing the window of implantation is initiated by progesterone exposure and not by systemic LH levels.

Our observation of women who had LH increase two days before progesterone rise having significantly higher BMI and significantly lower AMH values than women having both on the same day, would require further study to determine whether relying on progesterone levels can be particularly beneficial for women with high BMI and or decreased ovarian reserve. Using the progesterone rise as the benchmark for ovulation requires the availability of same day testing, results in increased costs, i.e., additional clinic visits and loss of working time as opposed to simple urinary LH testing at home. Whether these are justified by better synchronization of the embryo and the endometrium and higher live birth rates remains to be determined by **a** properly designed and adequately sized randomized controlled trial comparing NC FET based on LH vs. progesterone testing which would provide the definitive answer. The trial should include adequate numbers of women in different categories of BMI and AMH levels.

## Data Availability

The dataset supporting the conclusions of this article are available on request but have not been made available publicly.
